# Mentorship in otorhinolaryngology, a latent need^☆^

**DOI:** 10.1016/j.bjorl.2019.04.007

**Published:** 2019-05-20

**Authors:** Ricardo Vieira Teles Filho

**Affiliations:** Universidade Federal de Goiás (UFG), Faculdade de Medicina, Departamento de Cirurgia de Cabeça e Pescoço, Goiânia, GO, Brazil

*Dear Editor,*

We read the editorial “A call for mentorship in otolaryngology” with great interest.^1^ We strongly agree with the author when he says that “Mentorship is fundamental. The guidance, wisdom and benevolence of a more experienced colleague are very important and will guide a beginning career.” The English word “mentoring” (translated as *mentoria* or *tutoria* in Portuguese) originates from a character from Homer's Odyssey. Mentor was a friend of Ulysses who cared for his son Telemachus while his father returned from the Trojan War. Mentor, aided by the goddess Athena, was a practical guide to knowledge, and also a source of personal support for the young man. Today, in the presence of a high burnout rate and increased suicide rates in the medical setting, the issue is crucial.^2^ The mentorship should be initiated in the undergraduate years, with the purpose of helping the student choose an area of specialization and should persist throughout its duration.^3^ Especially in Otorhinolaryngology, a discipline that usually has little student exposure in the undergraduate curriculum, and a prevailing unawareness of the scope of this specialty in the great majority of medical schools, and also because it is a surgical specialty, with long learning curves mentorship is highly desirable. During the process of choosing a specialty, during mentors can show, the challenges, difficulties and scope of the specialty; during the residency, mentors can assist with the daily learning processes.^4^ We should indeed foster this practice in our services in order to encourage undergraduate students to evaluate and choose our area of specialty and facilitate residents’ learning process and skill development, guiding not only the theoretical and practical knowledge inherent to the specialty, but also being a source of personal support for the young otorhinolaryngologist as Mentor did in the Odyssey. By being the pioneer in this practice, Otorhinolaryngology will become an example for academic development and quality of life indexes of its residents and specialists.^5^

## Conflicts of interest

The author declares no conflicts of interest.
